# A Four-Channel Millifluidic Electrochemical Flow Reactor for Parallel Screening of Cathodically Active Microbial Communities

**DOI:** 10.3390/mi17060664

**Published:** 2026-05-27

**Authors:** Ting Xie, Olivia Gerhard, Steffen Schneider, Jialan Cao, Johann Michael Köhler

**Affiliations:** 1 Group for Physical Chemistry and Microreaction Technology, Institute for Chemistry and Bioengineering, Technische Universität Ilmenau, 98693 Ilmenau, Germany; ting.xie@tu-ilmenau.de (T.X.); olivia.gerhard@tu-ilmenau.de (O.G.); steffen.schneider@tu-ilmenau.de (S.S.); 2 Faculty of Engineering and Computer Science, Osnabrück University of Applied Sciences, 49076 Osnabrück, Germany

**Keywords:** millifluidic reactor, electrochemical screening, optical density monitoring

## Abstract

This study introduces a four-channel millifluidic microbial electrochemical reactor (mMER) designed for parallel screening of cathodically active microbial communities. This platform integrates electrochemical control with online optical density (OD) monitoring, enabling simultaneous tracking of biomass-associated OD development and current response in four separate culture systems. The system’s performance was initially validated through photometric calibration and biocompatibility tests, leading to the development of a standardized workflow for cathodic enrichment protocols of soil microbial communities. Using this system, a cathode potential of −0.4 V (vs. quasi-reference electrode) was identified as effective and applied to four different soil samples, revealing variations in their electrochemical responses. Based on combined OD and current measurements, a time-resolved ΔOD/ΔQ analysis was introduced to compare biomass-associated electrochemical response behavior during enrichment. This analysis revealed distinct temporal response characteristics among soil microbial communities, including early, delayed, and sustained responses observed under cathodic cultivation conditions. Open-circuit potential measurements, conducted after enrichment, provided additional electrochemical characterization of the enriched systems. Among all samples, the microbial community from a Neolithic earthwork site (Niedersickte, Germany, sample HB51) demonstrated the most stable relationship between cathodic current consumption and OD development during enrichment. The mMER platform offers a valuable tool for the comparative screening of electroactive microbial communities from complex environmental samples.

## 1. Introduction

Storing surplus energy is a key challenge in the energy transition. Renewable sources like wind and solar can produce more electricity than needed at times. Conversely, low wind or sunlight can lead to unmet demand. Traditional power plants, such as coal or nuclear, can also generate excess energy, but their output cannot be adjusted quickly. Therefore, local energy storage systems that can be charged and discharged rapidly are necessary [1,2,3]. Batteries offer a direct solution, but high-capacity electrochemical energy storage involves high costs and technical challenges [4,5]. Another option is to convert and store the excess energy as chemical energy using liquid or solid carriers, such as hydrogen, produced via water electrolysis. However, this process involves complex production methods [6,7].

Biological energy conversion presents a compelling alternative. In this process, the metabolic activity of living organisms is used to convert electrical or environmental energy into chemical energy that can be utilized. An example is the production of biogas from complex agricultural biomass, where methane-producing microorganisms play a key role [8,9]. Additionally, some microorganisms show direct or indirect electrochemical activity [10,11,12]. Recent research has mainly focused on anodically active bacteria, which can oxidize organic substrates and transfer electrons to the electrode, enabling bioelectrochemical fuel cells [13,14,15]. Conversely, cathodically active microorganisms can fix carbon dioxide or bicarbonate using electrons from the cathode in the absence of organic electron donors, producing energy-rich biomass [16,17,18]. Successful selection of these microorganisms requires cultivation under controlled cathodic conditions in a bioelectrochemical system.

Current research on bioelectrochemical reactors generally follows two approaches: scale-up or scale-down. Scale-up systems can achieve higher productivity and greater energy storage, but their limitations include long incubation times, relatively small electrochemically active surface areas, high consumable use, and complex operation [19,20,21,22,23]. Conversely, scale-down systems can enable high-throughput cultivation and multi-parameter analysis but face challenges related to electrode material costs and electrochemical efficiency. Their applications are mainly focused on well-characterized, highly electroactive model organisms, such as Shewanella and Geobacter [24,25,26,27]. Given these limitations, there is an urgent need for a screening electrochemical platform that combines the benefits of both approaches: ensuring sufficient biomass production, enabling systematic assessment of microbial electroactivity, and facilitating the discovery of novel, previously uncharacterized cathodically active microorganisms.

To meet this need, we developed a four-channel millifluidic Microbial Electrochemical flow Reactor (mMER) for potential-controlled, parallel screening of cathodically active microbial communities. The platform combines independent three-electrode systems with continuous medium circulation and online optical density monitoring. To ensure electrons are supplied solely by the cathode, microorganisms are cultivated in a bicarbonate-based inorganic medium. The system enables comparative enrichment of soil-derived microbial communities, quantitative assessment of potential-dependent biomass growth under controlled and comparable electrochemical conditions, and scalable screening of electroactive microorganisms from complex environmental samples.

## 2. Materials and Methods

### 2.1. Integrated mMER Platform

#### 2.1.1. Reactor Design

**Figure 1:**
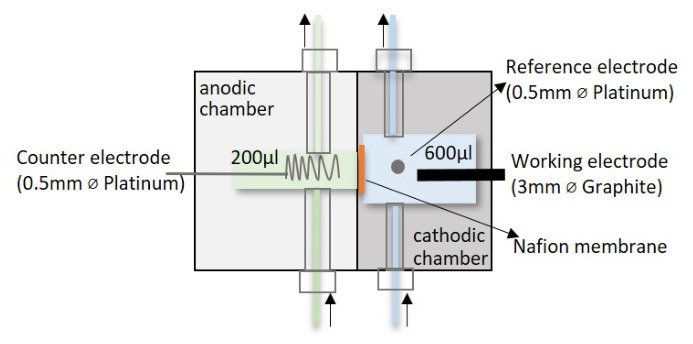
Schematic illustration of the mMER internal structure: a right cathode chamber (600 µL) with a 3 mm graphite working electrode and a 0.5 mm platinum wire quasi-reference electrode, separated from the left anode chamber (200 µL) by a Nafion membrane containing a 0.5 mm platinum wire coil counter electrode.

The mMER consists of miniaturized H-type cells fabricated from polyoxymethylene (POM) using precise drilling at the university workshop. Each reactor has two chambers—cathodic and anodic—separated by a Nafion^®^ N117 membrane (Ion Power GmbH, München, Germany) (Figure 1). The membrane was initially cut into circular pieces with a 1 cm diameter to fit the setup. It was then pretreated by soaking in 3% H_2_O_2_ at 80 °C for 1 h. After rinsing with deionized water, the circles were incubated in 0.5 M H_2_SO_4_ at 80 °C for 1 h, rinsed again, and sterilized by autoclaving in deionized water. The prepared membrane was placed between the chambers and secured with a 1 cm diameter elastic rubber gasket. The reactor halves were fastened with screws to ensure stability and a leak-proof seal. After inserting the electrodes, the chamber volumes were measured by pipetting electrolyte into each chamber. The anodic chamber holds 200 μL, while the cathodic chamber holds 600 μL.

#### 2.1.2. Electrode Configuration

Each reactor uses a three-electrode setup. The working electrode is a graphite rod (3 mm diameter, Tianwang Shimo, Dongguan, China), inserted 1 cm into the cathodic chamber, with an effective surface area of approximately 0.94 cm^2^. A platinum wire (0.5 mm diameter, R. Götze GmbH & Co. KG, Berlin, Germany), coiled to about 2 cm, functions as the counter electrode and is placed in the anodic chamber (Figure 1).

Due to the small size of the miniaturized reactor, using a standard Ag/AgCl reference electrode was impractical. Instead, a platinum wire (0.5 mm diameter) inserted about 2 mm into the cathodic solution served as a Quasi-Reference Electrode (QRE). Platinum was selected for its chemical stability under the experimental conditions and its common use as a QRE in microscale electrochemical systems where standard reference electrodes are challenging to implement [28].

It should be noted that a Pt QRE does not provide a fixed thermodynamic reference potential and may exhibit small variations depending on local electrochemical conditions. Therefore, all reported potentials are referenced to the Pt QRE and should be interpreted as relative.

To verify the stability of the cathode potential during long-term operations, the potential of the working electrode and the QRE were periodically checked using a multimeter. The measured drift remained below 5 mV over 65 h, indicating reproducible relative potential control during the experiment. This reflects the stability of the measurement setup rather than the absolute stability of the QRE. To ensure comparability between reactors, all measurements were taken with identical QRE setups, and potential drift was checked before each experiment.

The graphite working electrode was sealed with a matching 3 mm PTFE fitting. The reference and counter electrodes, along with the inlet and outlet connections, were attached using standard 1/16-inch PTFE fittings and ETFE tubing, ensuring leak-proof and stable operation under continuous flow conditions.

#### 2.1.3. Flow Circulation System

The system operated in a continuous closed-loop cycle. A peristaltic pump (12 V DC, 0.3 A, Kamoer, Shanghai, China) transferred the microbial suspension from an external culture bottle to the cathodic chamber at a steady flow rate of 1.5 mL min^−1^. This flow rate ensured continuous mixing while preventing excessive shear stress that could affect microbial activity. The flow rate was manually adjusted using the integrated speed control. Before the experiments, the flow rate was calibrated by measuring the pumped volume over a set time (1 min) with a pipette. After calibration, the pump setting remained fixed throughout each experiment.

The cathodic effluent flowed through an in-line optical flow cell connected to a photometer (Section 2.1.4) for continuous OD monitoring before returning to the same culture bottle, creating a recirculating loop. The cathodic circuit contained 20 mL of microbial suspension in a 100 mL culture bottle during cultivation.

The anodic chamber was circulated independently with 100 mL of electrolyte, creating a sufficiently large ionic reservoir to maintain electroneutrality. Since there are no microorganisms in the anodic circulation system, it does not include an extra optical detection unit. During the 3–4-day cultivation period, no medium exchange or replenishment was performed. This method prevented disturbances caused by medium replacement and ensured that any observed changes were solely due to microbial activity and electrochemical conditions.

All connections used PTFE tubing (1.6 mm OD and 1 mm ID), except for a small section of silicone tubing (3 mm OD and 1 mm ID) that was employed for the peristaltic pump to ensure proper functioning. In the cathodic loop, the inlet tubing was positioned below the liquid surface, while the outlet tubing was placed above the liquid level, creating a height difference between the inflow and return. External agitation (such as shaking) was not applied, as mechanical disturbances could interfere with electrochemical measurements, current stability, and optical density monitoring. Instead, the suspension was mixed in the culture flask by directing the returning flow onto the liquid surface, causing continuous droplet impact and surface perturbation. This passive mixing method maintained effective homogenization of the microbial suspension while ensuring stable measurement conditions.

Independent circulation of the cathodic and anodic compartments prevented cross-mixing while maintaining stable electrochemical and hydrodynamic conditions.

#### 2.1.4. Electrochemical Control and Online Monitoring

Electrochemical control was carried out using a Keithley 2400 SourceMeter (Keithley Instruments, Cleveland, OH, USA) operated in potentiostatic mode. For cathodic enrichment experiments, a constant potential was maintained throughout cultivation. The voltage was set to ±10 V with a current compliance of ±10 mA to manage initial current spikes from double-layer charging and transient processes. To prevent recording these artifacts, current measurement began 10 min after polarization, and the current was then recorded continuously at 1 Hz for 3–4 days.

Prior to the enrichment experiments, the open-circuit potential (OCP) was measured for 1 min after stabilization to establish baseline electrode conditions. After enrichment, OCP was measured again for 90 min to assess possible changes on the electrode surface (Section 3.7) or in the medium.

Optical density was monitored online with a custom-built four-channel photometric system equipped with 605 nm LEDs. Each flow cell used replaceable fluorinated ethylene propylene (FEP) tubing (1.6 mm OD and 1 mm ID, with a 1 mm path length), which minimized signal interference from biofilm formation. The use of replaceable tubing also allowed for periodic renewal between experiments if signal drift was observed.

The data acquisition and analysis were carried out using LabView software 2026. Simultaneous monitoring of electrochemical and optical data enables a direct correlation between current and microbial growth. Offline OD_605_ measurements were performed with a SPECORD 210 PLUS spectrophotometer (Analytik Jena GmbH, Jena, Germany) using a standard 1 cm path length cuvette.

### 2.2. Cultivation Conditions

#### 2.2.1. Medium Composition

To support cathodic microbial enrichment while ensuring that the cathode is the only electron donor for microbial metabolism, a modified M9 inorganic medium was designed to minimize alternative electron donors. Compared to conventional M9 medium, the concentrations of phosphate, calcium, and magnesium were reduced to minimize precipitation under electrochemical conditions. In additions, sodium bicarbonate and potassium nitrate were introduced as the primary inorganic carbon and nitrogen sources, respectively. The composition of the conventional M9 medium is provided in Table S5. The medium contained no added organic carbon sources, thereby promoting enrichment conditions dependent on cathodic electron availability.

A borax–phosphate buffer (Kolthoff buffer) was used to maintain a stable pH of 9 during electrochemical cultivation. For 50 mL of buffer, 41.5 mL of Na_2_B_4_O_7_·10H_2_O (0.05 M) was mixed with 8.5 mL of KH_2_PO_4_ (0.1 M). The resulting buffer solution was added to the cathodic medium at 7.15% (*v*/*v*). The slightly alkaline pH (9.0 ± 0.1) was selected to ensure a stable electrochemical environment and to exert selective pressure on the microbial community, encouraging electroactive microorganisms while limiting the growth of fast-growing heterotrophic populations.

Bulk salts, including K_2_HPO_4_ (0.001 M), NaCl (0.005 M), and KNO_3_ (0.002 M), were dissolved in deionized water and autoclaved to sterilize. After cooling to room temperature, filter-sterilized CaCl_2_·2H_2_O (0.01 mM), MgSO_4_·7H_2_O (0.25 mM), and trace element solution (SL10, 10 mL/L) were added. Sodium bicarbonate (15 mM) was separately filter-sterilized and added immediately before each experiment to minimize pH shifts caused by autoclaving. The medium pH before inoculation was 9.0 ± 0.1.

The anodic compartment held a supporting electrolyte of 7 mM K_2_HPO_4_, 3 mM KH_2_PO_4_, and 15 mM Na_2_SO_4_ (pH 7.3 ± 0.2). A simplified electrolyte was selected for the anodic side rather than the same complex medium as the cathodic chamber, as the Nafion membrane effectively separates the two compartments with minimal cross-mixing. The sodium sulfate concentration was adjusted to achieve a conductivity of approximately 4.5 mS cm^−1^, balancing the ionic strength between the cathodic and anodic chambers to ensure stable electrochemical conditions while preventing unnecessary consumption of medium components. After the experiment, the pH of the anodic electrolyte remained around 7.5, with negligible change from its initial value.

Unless otherwise stated, all chemicals and reagents were purchased from Merck (Darmstadt, Germany) or VWR (Radnor, PA, USA).

#### 2.2.2. Inoculum Preparation

Soil samples were air-dried and stored at room temperature before use. For inoculation, 0.5 g of dry soil (10% *w*/*v*) was suspended in 5 mL of phosphate-buffered saline (PBS) in a sealed 15 mL centrifuge tube and shaken for 2 h at room temperature. After shaking, the suspension was allowed to settle for 1 h. The suspension containing suspended vegetative cells and spores was then collected. Subsequently, 2 mL of this suspension was transferred to 18 mL of fresh medium, corresponding to a 10% (*v*/*v*) inoculation ratio.

Cycloheximide (CHX) was not added to suppress fungal growth because subsequent cultivation was performed in an inorganic medium lacking an organic carbon source, which naturally limits fungal and other heterotrophic microorganism growth. Additionally, adding CHX could introduce trace organic compounds that might interfere with maintaining bicarbonate as the sole carbon source in electrochemical cultivation.

#### 2.2.3. Pre-Cultivation

A short pre-cultivation period of 2–3 days was conducted before electrochemical enrichment to activate the dormant soil microbial community. During this phase, the medium was supplemented with 1 mM sodium lactate as a limited electron donor to stimulate metabolic activity in the otherwise nutrient-limited inorganic medium. Pre-cultivation was ended at the onset of growth—characterized by the first visible turbidity—well before reaching the exponential phase. At this point, the community was metabolically active but not yet enriched for fast-growing heterotrophs, maintaining community diversity for subsequent electrochemical selection. Lactate was not added during subsequent electrochemical experiments, ensuring that electrons during cathodic cultivation were supplied solely from the electrode. However, cell suspension of the pre-culture was directly transferred to the electrochemical system without washing to minimize disturbance to the microbial community. Therefore, residues of lactate inside the bioelectrochemical system cannot be ruled out.

### 2.3. Soil Samples

Five soil samples from various archeological and environmental sites across Germany were selected to represent different microbial community compositions for cathodic enrichment screening. For selected sites (Schöps and Jena, Inselplatz), detailed microbial community profiles have been reported previously [29,30,31]. All samples had pH values ranging from neutral to alkaline (7.0–8.3), making them suitable for electrochemical cultivation experiments. Conductivity levels varied widely between samples (52–2319 µS cm^−1^), reflecting their diverse geochemical origins. 16S rRNA gene sequencing revealed high microbial diversity across all samples, with sufficient sequencing depth (total reads between 57,000 and 203,000), enabling reliable community characterization and indicating a high potential for cathodic activity. Based on these qualities, the chosen soils served as a suitable basis for comparing cathodic enrichment behavior across different microbial inocula. An overview of sample locations and key properties is provided in Table 1.

### 2.4. Data Analysis

All raw data processing was conducted using Microsoft Excel and OriginPro 2025. To reduce noise and lessen the effect of short-term fluctuations caused by pumping cycles or minor temperature changes, current and online OD readings, recorded at 1 Hz, were averaged over 1 h intervals before further analysis. All subsequent calculations relied on these hourly averaged values.

To evaluate the consistency of the four online photometers during calibration (Section 3.2.1), the relative standard deviation (RSD) between the channels was calculated. For each methylene blue concentration, the RSD = (standard deviation divided by mean) × 100%. Using the linear regression tool in OriginPro, linearity was assessed, and the coefficients of determination (R^2^) for each calibration curve were computed.

Pearson correlation analysis was conducted to evaluate the relationship between online OD_605_ and cathodic current for the four soil bacterial communities (Section 3.5). Pearson correlation coefficients (r) were calculated using hourly averaged data from the start of enrichment to each specified time point. Positive r values indicate that microbial growth was linked to a decrease in cathodic current (less negative), while negative r values suggest that growth coincided with an increase in cathodic current (more negative). Values of 1 or −1 at later time points (e.g., 99 h) reflect the limited number of data points in the final interval. All calculations were performed with Microsoft Excel.

The relationship between OD development and charge transfer was evaluated using a ΔOD/ΔQ analysis (Section 3.6). Cumulative charge (Q, in µA·h) was determined by integrating the current over time using the trapezoidal rule.

$$Q=\sum_{i=1}^{n-1}\frac{I_{i}+I_{i+1}}{2}\times \Delta t$$

where *I_i_* and *I_i_*_+1_ are consecutive current measurements, and Δ*t* is the time interval (1 h after averaging). To distinguish temporal changes in biomass-associated electrochemical behavior during enrichment, an interval-based analysis was used:

$$\Delta OD/\Delta Q=\frac{{OD}_{t2}-{OD}_{t1}}{Q_{t2}-Q_{t1}}$$

Ratios were calculated for three intervals corresponding to different growth phases: 0–20 h (mid-exponential), 20–40 h (late exponential to early stationary), and 40–60 h (stationary phase). Values are reported as ×10^−4^ OD·µA^−1^·h^−1^ for clarity.

## 3. Results

### 3.1. System Architecture of the mMER Platform

**Figure 2:**
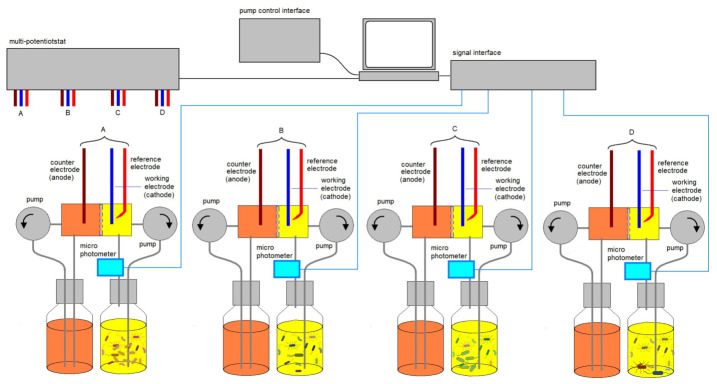
Schematic diagram of the four-channel millifluidic electrochemical reactor (mMER) platform. Panels A–D represent the four independent electrochemical flow channels, which have identical configurations and are operated in parallel. The diagram illustrates the overall system, including the four independent flow cells, four-channel potentiostat, multichannel peristaltic pump, online photometers, and external culture bottle.

The four-channel mMER platform was designed to enable parallel enrichment and comparative screening of cathodically active microbial communities under defined electrochemical conditions. As shown in Figure 2, the system includes four identical flow-loop channels operating in parallel. Each channel features a miniaturized three-electrode electrochemical flow cell (with working, counter, and reference electrodes), one channel of a multichannel peristaltic pump, one channel of a four-channel potentiostat, an online photometer for continuous optical density (OD) monitoring, and an external culture bottle connected via a closed recirculation loop. This modular setup allows for controlled, side-by-side comparison of different inocula or electrochemical conditions within a single experimental platform. Notably, this design enables the concurrent collection of electrochemical and biological data under identical conditions, reducing variability between samples.

During operation, the microbial suspension is continuously recirculated through the flow loop. The culture is pumped from the external bottle into the electrochemical cell, where a defined cathodic potential is applied to the working electrode to promote the enrichment of electrochemically responsive microorganisms. The effluent then passes through the in-line photometer for real-time OD measurement before returning to the culture bottle, where mixing occurs through the returning flow. This closed-loop configuration enables long-term cultivation while maintaining stable electrochemical and hydrodynamic conditions.

The recirculating flow-loop design offers several advantages compared to conventional static microscale cultivation systems. Continuous circulation facilitates the redistribution and release of gases (e.g., H_2_) generated during long-term cathodic polarization, thereby preventing local gas accumulation that may interfere with electrochemical measurements or mass transfer. At the same time, recirculation helps maintain stable pH and ionic conditions and reduces the accumulation of metabolic byproducts, which are common limitations in static microscale reactors [32]. The gentle flow conditions also minimize mechanical shear compared to shake-flask cultivation, providing a stable hydrodynamic environment suitable for electrochemical studies. In addition, the use of 1 mm ID tubing and compact flow cells enables operation with small total culture volumes (20 mL per channel, including the liquid volume inside the flasks), reducing reagent consumption while maintaining environmental stability.

By integrating electrochemical control with continuous optical monitoring, the mMER platform enables simultaneous recording of current, potential, and biomass-associated OD dynamics in four independent cultures over extended periods. This capability enables comparative analysis of the temporal relationship between OD development and electrochemical responses and provides an efficient platform for comparative screening of electrochemically responsive microbial communities.

### 3.2. Validation of the Integrated Measurement System

#### 3.2.1. Photometric Calibration

To validate the accuracy and consistency of the online OD measurements across the four channels, we conducted a photometric calibration using methylene blue as a model chromophore. The four photometers were operated in a parallel configuration using a common inlet manifold to ensure identical sample distribution. A peristaltic pump delivered stepwise varying concentrations of methylene blue (1–128 µM) at a constant flow rate, ensuring that all channels received the same sample simultaneously. Measurements were taken at 605 nm through FEP tubing, resulting in an effective optical path length of about 1 mm—roughly one-tenth of that of a standard 1 cm cuvette.

**Figure 3:**
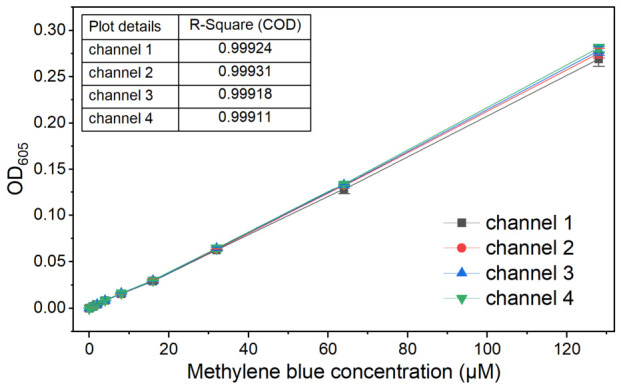
Photometric calibration of four online OD sensors at 605 nm. Absorbance versus methylene blue concentration (1–128 µM) for four channels operated in a parallel configuration using a common inlet manifold. All curves demonstrate excellent linearity (R^2^ > 0.99). Error bars (black) represent the standard deviation of three independent measurements (*n* = 3).

As shown in Figure 3, all four channels demonstrated excellent linearity across the entire concentration range (R^2^ > 0.99 for each channel). Additionally, the calibration curves were nearly overlapping, especially at concentrations of 4 µM and below. An analysis of inter-channel variability was then conducted, revealing a concentration-dependent pattern (Figure 3 inset; Table S1). At the lowest tested concentration (1 µM, OD_605_ ≈ 0.002), the relative standard deviation (RSD) between channels was 9.47%. At 2 µM (OD_605_ ≈ 0.004), the RSD decreased to 3.45%. As the concentrations increased further, the RSD continued to decrease, reaching approximately 1.5% within the range of 16–128 µM.

These results show that the four photometric channels have high linearity and low variability between channels, confirming their appropriateness for quantitative parallel monitoring. Importantly, variability decreased as signal intensity increased, suggesting that measurement uncertainty is mainly limited at very low OD values.

#### 3.2.2. Biological Validation of the Flow System

To determine whether continuous recirculation through the peristaltic pump and flow cells would negatively impact bacterial growth, we cultured Escherichia coli in LB medium within the mMER system under open-circuit conditions for 83 h. At the same time, a control culture was kept in a shake flask (100 rpm) at room temperature. Both cultures were inoculated at an initial OD_605_ of about 0.02 (measured with a 1 cm cuvette).

**Figure 4:**
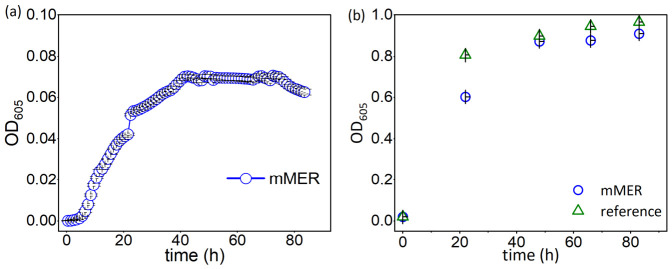
Biological validation of the flow system with *Escherichia coli* in LB medium. (**a**) Online OD_605_ profile of *E. coli* grown in the mMER system (1 mm path length). The curve represents hourly averaged values derived from continuous measurements recorded every second over an 83 h cultivation period. Error bars (black) represent the standard deviation of the raw measurements within each 1 h interval, reflecting temporal variability within the online signal. (**b**) Offline OD_605_ measurement (1 cm cuvette) for samples from the flow system (○) and shake flask reference (△). Data points represent the mean ± SD from triplicate experiments (error bars smaller than symbols).

As shown in Figure 4a, online monitoring within the mMER captured the growth dynamics in real time. The online OD_605_ data represent second-wise measurements, which were subsequently averaged into 1 h intervals for visualization. After an 8 h lag period, OD_605_ rises from 0.002 to 0.07 during the exponential growth phase (8–40 h), before stabilizing in the stationary phase.

For offline comparison, samples were taken from both the mMER and the shake flask at five time points (0, 22, 48, 66, and 83 h) and measured using a standard 1 cm cuvette spectrophotometer (Figure 4b). While the mMER system showed slightly lower OD values compared to the shake flask (e.g., 0.90 vs. 0.96 at 83 h, *p* < 0.001; Table S2), the overall growth kinetics remained remarkably consistent.

The observed difference in OD values likely results from two factors. First, the gravity-driven mixing and recirculation flow in the mMER system differ from the continuous stirring in a shake flask. This can lead to changes in the oxygen concentration within the medium and affect homogeneity, which influences microbial growth and results in a slightly lower density of suspended microorganisms. Second, additional differences may be due to the varying OD_605_ measuring units. Although Lambert-Beer law accounts for the varying thickness of measuring cells, it assumes smooth and parallel walls. Since the tubing walls bend, scattering effects are induced, impacting the OD_605_ readings. Importantly, both cultivation and measurement captured nearly identical growth kinetics, with an exponential phase between 8 and 40 h and a stationary phase at approximately 40 h.

These results show that continuous recirculation and flow conditions in the mMER system do not significantly hinder microbial growth, confirming the platform’s biocompatibility. Importantly, maintaining growth kinetics suggests that the system can consistently detect relative differences between samples, even if absolute OD values vary slightly.

### 3.3. Workflow for Cathodic Enrichment Experiments

**Figure 5:**
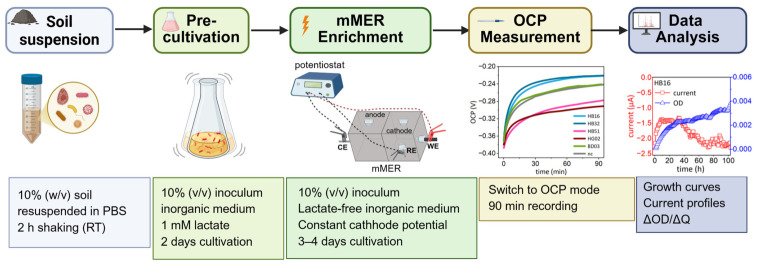
Schematic workflow for cathodic enrichment of soil microbial communities using the mMER platform, including sequential steps from soil suspension and pre-cultivation to electrochemical enrichment, open-circuit potential (OCP) monitoring, and data analysis (ΔOD/ΔQ).

Figure 5 illustrates the experimental workflow for cathodic enrichment of soil microbial communities using the mMER platform. Soil samples from covered layers were chosen based on differing geographical origins, archeological contexts, and physicochemical properties (pH, conductivity), detailed further in Section 2. The workflow was designed to selectively promote electroactive microorganisms while restricting the growth of fast-growing heterotrophs. Initial suspension in PBS preserves the native community structure by preventing premature growth [33]. A brief pre-cultivation in inorganic medium with low lactate levels activates microbial metabolism without major biomass buildup [34]. Any residual lactate (<0.1 mM) carried into the enrichment step was negligible and not enough to support heterotrophic growth, as verified by control experiments (Section 3.4).

This stepwise approach ensures a controlled transition from the native soil community to an electrochemically enriched system, thereby increasing the likelihood of enriching electrochemically responsive microorganisms. Using this workflow, we first examined the effect of cathode potential on enrichment with a single soil community (Section 3.4), then compared multiple soil samples under optimized conditions (−0.4 V, Section 3.5), conducted a time-resolved comparative ΔOD/ΔQ analysis to evaluate the relationship between OD development and charge transfer (Section 3.6), and performed post-enrichment electrochemical characterization using open-circuit potential (OCP) measurements (Section 3.7).

### 3.4. Effect of Cathode Potential on Enrichment of Electroactive Bacterial Communities

**Figure 6:**
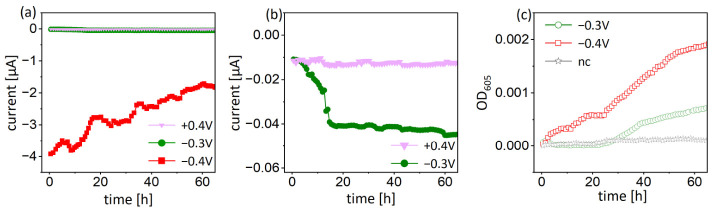
Effect of cathode potential on the enrichment of soil community BD03 over 65 h. (**a**) Current profiles at −0.4, −0.3, and +0.4 V. (**b**) Close-up of current profiles at lower current levels for −0.3 V and +0.4 V. (**c**) Online OD profiles for −0.3 V, −0.4 V, and the sterile control (nc).

In an inorganic medium where bicarbonate is the sole carbon source (representing dissolved CO_2_), microbial activity under these conditions is expected to depend primarily on cathodic electron availability. To find an appropriate cathode potential for enriching electroactive microorganisms, soil inoculum (BD03) was cultivated under three potentials: −0.3 V, −0.4 V, and +0.4 V (as an anodic polarization for comparison) for 65 h while monitoring current and online OD (Figure 6). Potential stability was confirmed independently using digital multimeter measurements.

A negative control without any microorganisms showed no OD increase over 65 h (Figure 6c), confirming the absence of contamination and indicating that the optical signal remains stable under non-biologically active conditions. A reference control experiment, where the same inoculum was incubated in lactate-free medium in a shake flask under identical conditions, showed no detectable growth by spectrophotometry. This confirms that any growth observed inside the bioreactor was not supported by residual carbon sources from the pre-culture.

At −0.4 V, a significant cathode current was observed immediately after polarization, starting at −4 µA and gradually decreasing to −1.8 µA over 65 h (Figure 6a). The observed current profile is consistent with cathodic electrochemical responses under the applied cultivation conditions. Together with the lack of growth in the shake-flask control, this supports the interpretation that cathodic polarization contributed to the observed biomass-associated OD development at −0.4 V.

In contrast, the current remained near baseline levels over 65 h at −0.3 V, ranging from −0.01 to −0.045 µA. Similar results were obtained at +0.4 V, where the current remained approximately constant at −0.012 µA throughout the experiment (Figure 6b).

The corresponding OD value curves shown in Figure 6c support previous observations for the applied negative potentials. At a potential of −0.4 V, the OD value increased to 0.002 after 65 h, suggesting biological activity under cathodic conditions. In contrast, at −0.3 V, only a minor OD increase (~0.0007) was observed, suggesting limited biomass-associated optical changes under these conditions. Conversely, a notable increase in OD was observed at +0.4 V, reaching approximately 0.0025 after 65 h despite the absence of cathodic current. Since the shake-flask control remained clear, the origin of this effect could not be conclusively identified and may involve multiple biological or electrochemical factors.

Based on the overall correlation between cathodic current and biomass development at −0.4 V, this potential was selected for all subsequent comparative experiments (see Section 3.5).

### 3.5. Cathodic Enrichment of Soil Microbial Communities at −0.4 V

**Figure 7:**
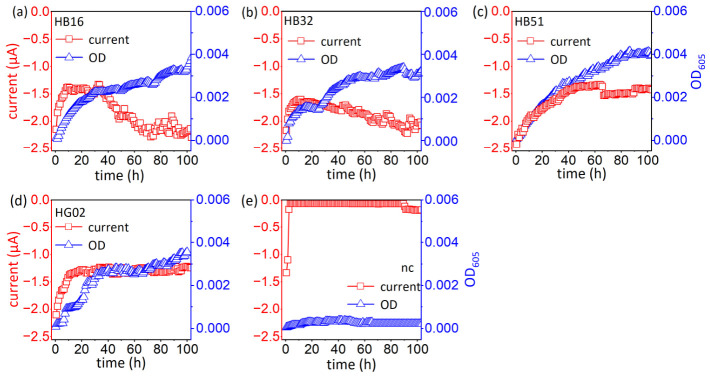
Cathodic enrichment of five soil communities at −0.4 V for 100 h. Current (red lines) and online OD (blue lines) profiles for soil samples: (**a**) HB16, (**b**) HB32, (**c**) HB51, (**d**) HG02, and (**e**) sterile control (medium only, no inoculum). Dual y-axes display current (left) and OD (right) for each panel.

To assess the mMER platform’s ability to distinguish different microbial inocula, we enriched five samples under identical conditions at −0.4 V for 100 h: four soil communities (HB51, HB32, HB16, HG02) and a sterile control (nc, medium only). Current and online OD were continuously monitored (Figure 7).

All four soil communities showed initial cathodic currents between −2.4 and −2.1 µA after polarization (Figure 7a–d). However, their current trends diverged after the first 10–20 h. HB51 experienced a positive current shift that stabilized at −1.4 µA after 40 h, while HG02 stabilized earlier, after only 10 h. In contrast, HB16 and HB32 displayed an initial positive current shift followed by a gradual decrease toward more negative values during later cultivation stages. For HB16, the current peaked at approximately −1.4 µA during the first hours and remained stable until 40 h before decreasing to −2.2 µA at 100 h. HB32 exhibited a similar pattern (see Figure 7b): after initially rising to −1.6 µA, the current starts to decline at 20 h, reaching −2.0 µA at 100 h. These differences in current evolution suggest distinct current response patterns among the soil-derived microbial communities under cathodic conditions. Possible contributing factors may include differences in microbial attachment behavior, community activity, or mass transport conditions at the electrode interface. However, the current data alone do not allow direct conclusions regarding biofilm formation or electrode colonization dynamics.

The sterile control group (nc) showed a different curve (Figure 7e), starting with an initial current of −1.5 µA that quickly dropped to −0.05 µA within 5 h and stayed at this baseline for the rest of the experiment. This current is due to capacitive effects that occur after the measurement begins. Since there are no redox reagents, the system quickly reaches electrochemical equilibrium, and no current flows. The OD value remained near zero (≈0.0002), confirming the absence of contamination and indicating the system’s background signal.

All four soil communities reached a relatively stable OD range between 40 and 60 h, with final OD values of 0.003–0.004 (Figure 7a–d). Distinct temporal OD dynamics were observed among the different microbial communities across the parallel channels. Since the measured OD values remained comparatively low due to the short optical path length of the online detection system, additional validation experiments using defined *E. coli* cell concentrations were performed to evaluate the biological relevance of the detected OD signals. The validation experiments demonstrated a clear correlation between mMER online OD signals, spectrophotometric OD measurements, and cell density, supporting that OD values within the observed range correspond to biologically relevant biomass levels (Figure S1). The measured OD signals should primarily be interpreted as relative biomass-associated optical changes rather than absolute biomass concentrations. Surface-associated biomass on electrodes or tubing walls may additionally influence the measured OD signals.

Analysis of the relationship between current and OD values in each sample revealed different patterns. For HB16, the OD value increased linearly from 0 to 40 h, then entered a slower growth phase. Notably, the start of the stabilization phase at 40 h coincided with the beginning of the current’s negative shift from −1.4 µA to −2.2 µA, indicating a temporal association between OD development and current dynamics during the later cultivation stages. For HB32, the OD value rose quickly within 0–10 h (reaching 0.0015), remained stable until 20 h, then increased again, reaching 0.0025 at 40 h, before entering a slow growth phase. Its current began shifting negatively at 20 h, coinciding with the second increase in OD. For HB51, the OD value kept increasing throughout the experiment without a clear stabilization phase, reaching 0.004 at 100 h, while its current stabilized at −1.4 µA after 40 h and stayed constant. For HG02, the OD reached a stable level (0.003) at 40 h, while its current stabilized at −1.2 µA at 10 h.

Pearson correlation analysis confirmed differences in the relationship between OD values and current for the four soil communities, and the correlation strength and pattern changed over time (Table S3). These distinct current trajectories indicate fundamentally different electrochemical behaviors among the different soil bacterial communities. However, current and OD alone do not fully capture the efficiency of electron utilization, requiring further quantitative analysis (ΔOD/ΔQ, Section 3.6).

### 3.6. Time-Resolved Analysis of ΔOD/ΔQ During Cathodic Enrichment

**Figure 8:**
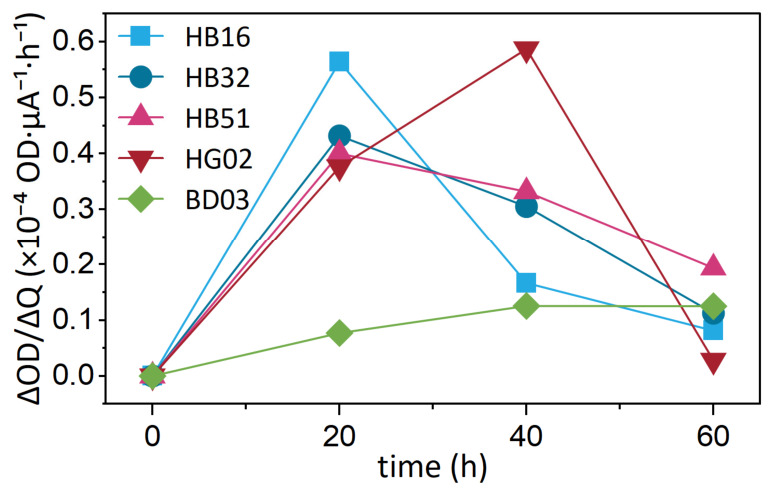
Time-resolved (ΔOD/ΔQ) analysis of enriched microbial communities. ΔOD/ΔQ values were calculated for three periods corresponding to the mid-exponential (0–20 h), late-exponential/early-stationary (20–40 h), and stationary (40–60 h) phases using the interval method. Each line represents a soil community enriched at −0.4 V: HB51, HB32, HB16, HG02 (Section 3.5), and BD03 (Section 3.4).

To further compare the electrochemical performance of soil communities enriched at −0.4 V, we analyzed the relationship between OD development and charge transfer using a time-resolved ΔOD/ΔQ approach. This analysis included five soil microbial communities: HB51, HB32, HB16, and HG02 (Section 3.5), and BD03 (Section 3.4). The ΔOD/ΔQ ratio was calculated over three intervals corresponding to key growth phases: 0–20 h (mid-exponential), 20–40 h (late exponential to early stationary), and 40–60 h (stationary phase) (Figure 8). This interval-based analysis allows comparison of temporal differences in biomass-associated electrochemical behavior between microbial communities.

The ΔOD/ΔQ relationship varied significantly across both growth phases and microbial communities (Figure 8), reflecting differences in the temporal coupling between OD development (ΔOD) and charge transfer (ΔQ). During the mid-exponential phase (0–20 h), most communities exhibited their highest ΔOD/ΔQ values. During this phase, microbial activity and suspended biomass development increased rapidly, resulting in relatively large ΔOD values, while cumulative charge transfer remains comparatively low. Consequently, the ΔOD/ΔQ ratio was maximized. Among the communities, HB16 showed the highest ΔOD/ΔQ value (0.56 × 10^−4^ OD·µA^−1^·h^−1^), followed by HB32 (0.43 × 10^−4^ OD·µA^−1^·h^−1^), HB51 (0.40), and HG02 (0.38). In contrast, BD03 displayed substantially lower values (0.078).

During the next phase (20–40 h), the overall ΔOD/ΔQ values decreased. This change reflects a shift in growth dynamics: OD development slowed, leading to a smaller ΔOD (OD_40_ − OD_20_), while charge transfer continued, resulting in a larger ΔQ (Q_40_ − Q_20_). Consequently, the ΔOD/ΔQ ratio decreased. Despite this overall trend, clear differences between communities remained observable. HG02 reached the highest ΔOD/ΔQ value (0.59), reflecting continued OD development under relatively stable current conditions during the 20–40 h interval (Figure 7d). Meanwhile, HB16 (0.17) and HB32 (0.31) showed significant decreases, whereas HB51 remained relatively stable at 0.33. BD03 increased gradually to 0.13, suggesting progressive adaptation to the electrochemical environment.

During the stationary phase (40–60 h), ΔOD/ΔQ values reached their lowest levels across all communities. OD development largely plateaued, resulting in minimal ΔOD (OD_60_ − OD_40_), while charge accumulation continued, further decreasing the ΔOD/ΔQ ratio. HB51 maintained the highest value (0.19), followed by BD03 (0.13), with HB32 (0.11), HB16 (0.08), and HG02 (0.027) showing significant declines.

These differences suggest distinct temporal patterns in growth-associated electrochemical activity among these microbial communities. HB16 and HB32 exhibited strong activity during the early enrichment phase, followed by a gradual decline over time. In contrast, HG02 reached its highest ΔOD/ΔQ values during the later enrichment phase, indicating continued OD development relative to charge transfer over time. BD03 displays a gradual increase in ΔOD/ΔQ values over time, suggesting gradual adaptation to the electrochemical environment. Meanwhile, HB51 maintains relatively steady ΔOD/ΔQ values throughout all growth phases. Although it does not reach the highest peak values, its comparatively stable behavior suggests a sustained, relatively stable relationship between OD development and charge transfer over time.

It should be noted that the ΔOD/ΔQ analysis is based on suspended optical density and therefore primarily reflects suspended biomass rather than total biomass in the system. As enrichment proceeds, a fraction of microbial biomass may be associated with surfaces, which is not captured by ΔOD measurements. This may lead to an underestimation of total biomass when biofilm formation occurs. Accordingly, the ΔOD/ΔQ ration should be interpreted as a comparative metric between different communities under identical conditions, rather than an absolute measure of biomass yield, electron conversion efficiency, or temporal biomass evolution within a single culture. Although the same reactor configuration was used for all communities, differences in biofilm-forming tendency may contribute to community-specific variation in the measured ΔOD/ΔQ values.

Despite these limitations, the method remains effective for identifying microbial communities with distinct electrochemical activity patterns in a parallelized system. The mMER platform enables such comparative screening of electrochemically responsive microbial communities under controlled and comparable operational conditions. To further characterize electrode-associated electrochemical changes after enrichment, the electrochemical state of the electrodes was later examined using open-circuit potential (OCP) measurements.

### 3.7. Open-Circuit Potential (OCP) After Enrichment

To evaluate how the enriched communities had altered the initial electrode potential, we measured OCP for 90 min immediately after disconnecting the potentiostat. OCP is the potential of the working electrode measured against a reference electrode when no external potential is applied. It reflects the combined electrochemical state of the electrode–medium interface after polarization has ceased. More negative OCP values indicate a more reducing environment state, which may be influenced by microbial activity, surface-associated growth, and electrode conditioning effects [35,36]. For comparison, OCP was also recorded before polarization at Day 0 (Table S4).

**Figure 9:**
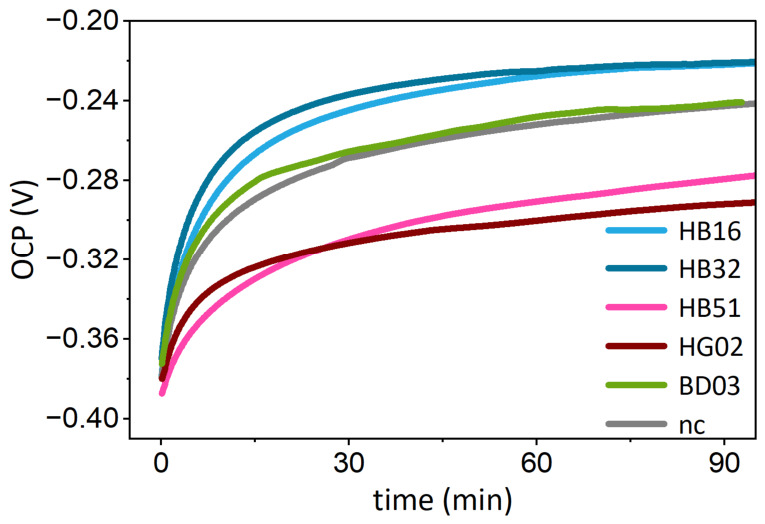
Open-circuit potential (OCP) measurements after enrichment at −0.4 V. OCP was recorded for 90 min immediately after disconnecting the potentiostat for five soil communities and a sterile control (nc). The Y-axis range (−0.2 to −0.4 V) was chosen to emphasize differences in potential recovery behavior among the different samples.

Before enrichment (Day 0), all electrodes exhibited similar OCP values ranging from −0.09 to −0.009 V (Table S4), indicating minor variations in the initial electrochemical state across channels, likely due to differences in the initial electrode surface conditions after assembly. Interestingly, after cathode enrichment was stopped (0 min), all samples showed nearly identical OCP values around −0.38 V (Figure 9), demonstrating that the applied −0.4 V polarization strongly influenced the electrode potential during enrichment. Over the next 90 min, all potentials moved toward more positive values as the electrodes relaxed from their polarized state. In the sterile control (nc), where microbes are absent, this relaxation likely reflected electrochemical equilibration within the abiotic system. In inoculated systems, the relaxation behavior may additionally have been influenced by biological and electrochemical changes occurring at the electrode interface during enrichment. Possible contributing factors include changes in media composition caused by electrochemical processes during cultivation, as well as electrode-associated biological or electrochemical effects established during enrichment.

After 90 min, distinct patterns appeared. HB51 and HG02 maintained significantly more negative potentials (−0.28 V and −0.29 V, respectively) than the sterile control (−0.24 V), indicating differences in the post-polarization electrochemical state between the enriched systems and the sterile control. These observations may reflect differences in electrode-associated processes or redox conditions established during enrichment. However, the underlying mechanisms cannot be directly resolved from OCP measurements alone. Nevertheless, the observed OCP trends are qualitatively consistent with the different electrochemical behaviors described in Section 3.6.

In contrast, HB16 and HB32 relaxed more quickly, reaching −0.22 V at 90 min, indicating a faster relaxation toward the baseline electrochemical state after polarization stopped. This behavior is qualitatively consistent with their earlier transient current response patterns observed in Section 3.6. BD03 remained close to the sterile control throughout the relaxation period, showing minimal influence on the electrochemical state of the system.

These observed OCP trends show qualitative agreement with the differences in electrochemical behavior identified through the ΔOD/ΔQ analysis. Communities that exhibited more sustained current and ΔOD/ΔQ trends (e.g., HB51 and HG02) also maintained more negative OCP values during post-polarization relaxation, whereas communities with earlier and more transient response patterns (HB16 and HB32) showed a faster return toward the baseline electrochemical state.

It should be noted that OCP measurements represent the combined electrochemical state of the electrode–medium interface and do not directly identify the underlying mechanisms. Therefore, the observed differences should be seen as indicative of system-wide electrochemical behavior rather than conclusive evidence of specific processes like biofilm formation or electron storage.

Nevertheless, the qualitative agreement between time-resolved efficiency trends and post-polarization OCP behavior further supports the applicability of the mMER platform for capturing complementary biological and electrochemical responses during comparative screening of electroactive communities.

## 4. Discussion

This study demonstrates the successful development of a four-channel mMER platform capable of parallel electrochemical cultivation with concurrent monitoring of biomass-associated OD changes and current response. Calibration verified that the system provides consistent and comparable OD measurements across channels, ensuring that observed differences in OD dynamics mainly reflect biological variation rather than absolute quantification of bias.

Continuous circulation did not significantly alter overall cultivation behavior compared to traditional shake-flask cultivation, although small differences in absolute OD values were seen. These differences are probably due to hydrodynamic conditions, surface-associated cell interactions within the tubing system, and optical scattering effects caused by the curvature of the tubing wall. Since the main goal of this study was comparative analysis of the temporal relationship between OD development and electrochemical responses rather than absolute biomass quantification, these minor deviations are considered acceptable.

The comparatively low OD values observed during cathodic enrichment likely reflect the intrinsically low-biomass nature of electrochemical cultivation under inorganic and low-substrate conditions rather than insufficient system sensitivity. In contrast to heterotrophic cultivation in nutrient-rich media, cathodic enrichment relies on energetically constrained electron uptake processes and inorganic carbon assimilation, which are generally associated with slower growth rates and lower suspended biomass formation. Therefore, small but reproducible OD changes may still contain biologically relevant information, particularly when interpreted together with electrochemical parameters.

The introduction of a low-substrate pre-cultivation step improved enrichment efficiency by reducing lag phases and activating dormant microorganisms. This preconditioning may facilitate the transition to bioelectrochemical growth by encouraging early metabolic activity. The delayed growth seen under electrochemical conditions further indicates that microbial communities need physiological adaptation before they can adapt to cathodic cultivation conditions as an energy source, emphasizing the importance of metabolic flexibility during enrichment processes.

Applied cathode potential was identified as a key factor influencing microbial electrochemical response behavior. More negative potentials were associated with increased cathodic current and stronger OD development, which may reflect enhanced thermodynamic favorability for electrode-associated redox processes under cathodic conditions [37,38]. Increased electron availability at more negative potentials may support microbial metabolism under inorganic carbon conditions [39,40]. In contrast, the lack of cathodic current at positive potentials confirms that electron uptake is energetically unfavorable, although residual growth indicates the presence of alternative metabolic pathways or internally stored substrates.

Distinct current–OD relationships observed across different microbial communities indicate diverse electrochemical activity patterns, potentially reflecting differences in electrochemical response behavior, community adaptation dynamics, and surface-associated growth characteristics [41]. To capture these differences in electrochemical response behavior, the ΔOD/ΔQ ratio was introduced as a comparative metric linking biomass-associated optical changes with charge transfer under identical conditions. Although the parameter does not represent a direct biomass yield coefficient, it provides insight into apparent electron-to-biomass conversion behavior and enables comparison of distinct electrobiological response patterns among microbial communities. The usefulness of this approach lies less in absolute quantification and more in identifying temporal differences in electron utilization behavior during enrichment. Nevertheless, interpretation within a single culture remains limited, particularly at later cultivation stages when surface-associated growth may contribute to underestimation of total biomass due to reliance on suspended OD measurements [42].

Complementary OCP measurements offered additional insights into the post-polarization electrochemical state of the system. Communities showing sustained or higher ΔOD/ΔQ values also demonstrated more negative OCP values after polarization stopped. However, OCP reflects the combined response of the electrode–biofilm–medium system and should therefore be viewed as an indicator of system-level electrochemical behavior rather than direct evidence of specific processes such as biofilm formation or electron storage. Nonetheless, the alignment between ΔOD/ΔQ trends and OCP behavior supports the robustness of the comparative analysis.

Overall, combining current monitoring, online OD measurement, ΔOD/ΔQ analysis, and OCP characterization creates a multi-parameter framework for evaluating microbial electroactivity. This integrated approach enhances screening reliability and enables functional comparison of electroresponsive behaviors among complex microbial communities, thereby supporting more informed optimization and selection strategies for electrochemical enrichment workflows.

Despite these advances, several limitations need to be considered. The lack of biological replicates reduces statistical robustness and should be addressed in future studies. Additionally, the complexity of soil microbial communities makes mechanistic interpretation difficult, as multiple bioelectrochemical processes may occur simultaneously. Finally, the 90 min OCP relaxation period might not fully capture long-term electrode recovery dynamics; longer monitoring could provide deeper insights into electrode–microbe interactions. Tackling these limitations will improve the applicability of the mMER platform for systematic investigation of electrochemically responsive microbial communities.

## 5. Conclusions

In summary, a four-channel µMER platform was developed for parallel electrochemical cultivation and real-time monitoring of current and optical density, allowing comparative evaluation of electrochemical activity and biomass-associated OD dynamics. The results show that both the applied potential and the source of the microbial community strongly influence electrochemical behavior and enrichment dynamics.

By combining time-resolved ΔOD/ΔQ analysis with OCP measurements, distinct temporal electrochemical response patterns were identified among soil-derived microbial communities. Notably, the HB51 community demonstrated comparatively stable coupling between OD development and charge transfer throughout the enrichment process, indicating sustained electrochemical activity behavior under cathodic conditions.

Overall, the mMER platform offers an effective and versatile tool for comparative screening of electrochemically responsive microbial communities from complex environmental samples. Its capability to capture both biological and electrochemical response patterns provides valuable insights into microbial enrichment behavior under controlled electrochemical conditions and supports future optimization of bioelectrochemical cultivation systems.

## Figures and Tables

**Table 1:** Overview of soil sample locations and physicochemical properties.

Sample	Location	Coordinates ^1^	pH	Conductivity (µS cm^−1^)	Total Reads ^2^
BD03	Burgdorf, Lesse	4379,442/5781,791	7.00	52.3	133,069
HB16	Schöps	4471,400/5633,000	7.44	285.3	57,035
HB32	Jena, Inselplatz	4471,371/5643,897	8.09	673.3	203,556
HB51	Niedersickte	4406,838/5787,100	8.08	74.2	104,819
HG02	Golmsdorf, Kirche	4476,725/5648,542	8.31	2319	72,989

^1^ Coordinates are given in the Gauss–Krüger coordinate system and represent approximate sampling locations within the archeological sites. ^2^ Total reads obtained from 16S rRNA gene sequencing of the original soil samples.

## Data Availability

The data supporting this study’s findings are available from the corresponding author upon request.

## Supplementary Materials

The following supporting information can be downloaded at: https://www.mdpi.com/article/10.3390/mi17060664/s1, Table S1: Inter-channel variability of online OD measurements at 605 nm. Table S2: Comparison of optical density (OD_605_) of *E. coli* growth between the mMER system and shake flask reference. Table S3: Time-resolved Pearson correlation coefficients between online OD_605_ and current. Table S4: Open-circuit potential (OCP) values before and after cathodic polarization enrichment. Table S5: Composition of conventional M9 medium stock solutions and preparation of 1× M9 medium used as reference for medium modification. Table S6: Raw calibration data used for determination of the empirical mMER OD correction factor. Figure S1: Calibration of the mMER online optical density measurements against conventional spectrophotometric OD measurements using defined *E. coli* suspensions.
