# Supplementary material for: A Four-Channel Millifluidic Electrochemical Flow Reactor for Parallel Screening of Cathodically Active Microbial Communities
Source: Micromachines (Basel). 2026 May 27;17(6):664. doi: 10.3390/mi17060664 (PMC13303757; doi:10.3390/mi17060664)
Supplement: Supplementary file 1 [file micromachines-17-00664-s001.zip › micromachines-4276732-supplementary.pdf]

Table S1. Inter-channel variability of online optical density (OD_{605}).

Concentration (μM)	Online OD_{605} 1 [i]	Online OD_{605} 2	Online OD_{605} 3	Online OD_{605} 4	Mean ± SD [ii]	RSD (%)
1	0,002	0,002	0,0024	0,0023	0.0020 ± 0.0002	9.47
2	0,0041	0,004	0,004	0,0043	0.004 ± 0.00014	3.45
4	0,0084	0,0083	0,0082	0,0088	0.008 ± 0.0003	3.12
8	0,0155	0,0155	0,016	0,0165	0.015 ± 0.0005	3.01
16	0,029	0,03	0,0295	0,0302	0.030 ± 0.0005	1.81
32	0,0625	0,0635	0,064	0,0645	0.064 ± 0.0009	1.34
64	0,1285	0,1322	0,133	0,134	0.13 ± 0.002	1.82
128	0,269	0,2755	0,278	0,281	0.28 ± 0.005	1.85

[i] Online optical density (OD_{605}) values were measured using four parallel photometers, connected in series and integrated in the mMER platform, with methylene blue at the indicated concentrations.

[ii] Mean ± standard deviation (SD) of four parallel photometric channels; RSD, relative standard deviation.

Table S2. Comparison of optical density (OD_{605}) of *E. coli* growth between the mMER system and shake flask reference.

Time (h)	Condition	OD_{605} 1 [i]	OD_{605} 2	OD_{605} 3	Mean ± SD	P-value [ii]	Significance
0	mMER	0.021	0.020	0.019	0.020 ± 0.001	0.64	ns
	reference	0.02	0.021	0.02	0.020 ± 0.001		
22	mMER	0.601	0.605	0.603	0.603 ± 0.002	2.7e-8	***
	reference	0.809	0.805	0.806	0.807 ± 0.002		
48	mMER	0.87	0.874	0.873	0.872 ± 0.002	0.0001	***
	reference	0.896	0.899	0.90	0.898 ± 0.002		
66	mMER	0.877	0.879	0.876	0.877 ± 0.0015	6.8e-7	***
	reference	0.944	0.947	0.945	0.945 ± 0.0015		
83	mMER	0.91	0.909	0.908	0.909 ± 0.001	1.9e-6	***
	reference	0.966	0.967	0.963	0.965 ± 0.001		

[i] OD_{605} values were measured using a standard 1 cm path length cuvette. Samples were collected from mMER and shake-flask cultures at the indicated time points. Data are presented as mean ± standard deviation (SD) of three independent measurements (n = 3).

[ii] P-values were calculated using Student's t-test (two-tailed, equal variance) in Microsoft Excel. *ns: not significant ($p > 0.05$); ***$p < 0.001$

Table S3. Time-resolved Pearson correlation coefficients [i] between online OD_{605} and current.

Soil sample	0-20h	0-40h	0-60h	0-80h	0-99h
HB16	-0,82	-0,66	0,004	-0,10	1
HB32	-0,79	-0,73	-0,42	-0,04	1
HB51	0.53	-0.47	-0.15	0,46	1
HG02	0,18	0,26	0,1	0,56	-1
NK	0,84	0,85	0,07	0,13	1

[i] Pearson correlation coefficients (r) between online OD_{605} and current were calculated using hourly-averaged data from the start of enrichment to each indicated time point. Positive r values indicate that microbial growth was associated with a decrease in cathodic current (less negative), whereas negative r values indicate that growth coincided with an increase in cathodic current (more negative). Values of 1 or -1 at 99 h reflect the limited number of data points in the final interval.

Table S4. Open-circuit potential (OCP) [i] values before and after cathodic polarization enrichment.

Sample	Day 0 (V)	0 min after polarization (V)	90 min after polarization (V)
HB16	-0.042	-0.38	-0.22
HB32	-0.020	-0.37	-0.22
HB51	-0.009	-0.39	-0.28
HG02	-0.090	-0.38	-0.29
BD03	-0.022	-0.37	-0.24
nc	-0.082	-0.38	-0.24

[i] OCP values (V vs. quasi-reference electrode) measured before enrichment (Day 0), immediately after cathodic polarization, and after a 90 min relaxation period. Data reflect the electrochemical response of each microbial community following cathodic enrichment in the mMER platform.

Table S5. Composition of conventional M9 medium stock solutions and preparation of 1× M9 medium used as reference for medium modification.

(A) M9 5× stock solution (per liter)

Compound	Amount
$Na_2HPO_4 \cdot 2H_2O$	42.5 g
KH_2PO_4	15 g
NH_4Cl	5.0 g
NaCl	2.5 g

(B) Preparation of 1× M9 medium (final volume: 1 L)

Compound	Volume added
M9 5× stock solution	200 mL
100 mM $CaCl_2$ stock solution	1 mL
Trace element solution (100×)	10 mL
1 M $MgSO_4$ stock solution	1 mL

Depending on the experimental requirements, different carbon sources may be added before adjusting the final volume to 1 L with deionized water.

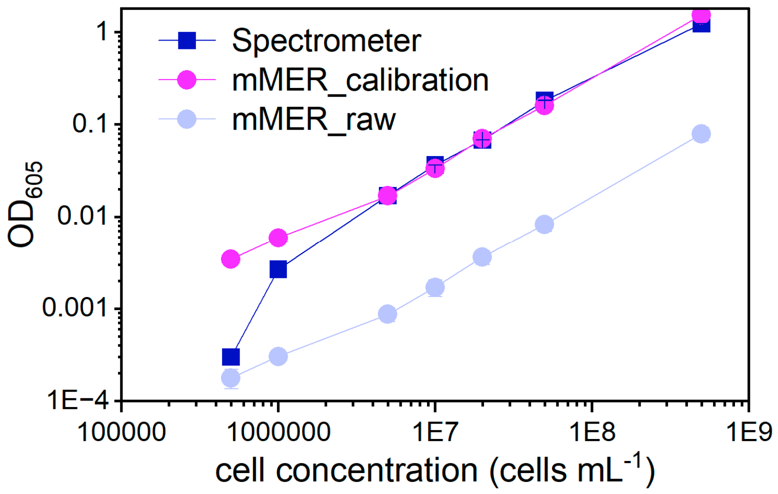

Figure S1. Calibration of the mMER online optical density measurements against conventional spectrophotometric OD measurements using defined *E. coli* suspensions. Serial dilutions of *E. coli* ranging from 5×10^5 to 5×10^8 cells mL^{-1} in PBS were sequentially measured using the mMER online optical setup (1 mm optical path length) and a conventional spectrophotometer at 605 nm (1 cm path length). An empirical correction factor of 19.43 (Table S7) was determined and applied to the raw mMER OD values. Raw mMER OD values of 0.003–0.004 corresponded to corrected spectrophotometric OD values of approximately 0.058–0.078 after calibration. Both axes are displayed on logarithmic scales.

Table S6. Raw calibration data used for determination of the empirical mMER OD correction factor.

Cell density (cells mL^{-1})	Spectrometer OD_{605}	Raw mMER OD_{605}	Calibration factor	Included in calibration
5×10^5	0.0003	0.0002	1.69	no
1×10^6	0.0027	0.0003	8.79	no
5×10^6	0.017	0.0009	19.61	yes
1×10^7	0.0363	0.0017	21.28	yes
2×10^7	0.0679	0.0036	18.7	yes
5×10^7	0.1822	0.0083	22.01	yes
5×10^8	1.2317	0.0792	15.54	yes

The empirical mMER OD correction factor was calculated as the arithmetic mean of the five calibration ratios included within the linear measurement range (5×10^6 – 5×10^8 cells mL^{-1}), resulting in an average factor of 19.43.
